# Histomorphometric Comparison between Two Types of Acellular Dermal Matrix Grafts: A Mini Pig Animal Model Study

**DOI:** 10.3390/ijerph18083881

**Published:** 2021-04-07

**Authors:** Javier Aragoneses, Ana Suárez, Cinthia Rodríguez, Juan Manuel Aragoneses

**Affiliations:** 1 Department of Medicine and Medical Specialties, Faculty of Health Sciences, University of Alcalá, 28871 Alcalá de Henares, Spain; javias511@gmail.com; 2 Department of Preclinical Dentistry, School of Biomedical Sciences, Universidad Europea de Madrid, 28670 Villaviciosa de Odón, Spain; 3 Department of Dentistry, Federico Henriquez y Carvajal University, 11005 Santo Domingo, Dominican Republic; cinthiagarabitos@gmail.com; 4 Faculty of Dentistry, Universidad Alfonso X El Sabio, 28961 Villanueva de la Cañada, Spain; jmaragoneses@gmail.com

**Keywords:** animal model, acellular dermal matrix graft, angiogenesis, keratin, wound healing

## Abstract

Acellular dermal matrix grafts (ADMG) have been used as soft tissue graft substitutes for autografts in periodontal plastic surgical procedures. They have benefits like avoiding a second surgical site and patient morbidity that have been associated with autografts, but there is limited evidence available on their tissue response and wound healing process. This histomorphometric animal model study was carried out in mini pigs and it aimed to compare the two types of ADMG materials of porcine derivative with a control group through observation of parameters like epithelial and Keratinized layer thickness, angiogenesis, cellularity, matrix resorption, and inflammatory infiltrate. The surgical technique involved punctures on the edentulous areas stripping the epithelial tissue and exposing the underlying connective tissue, placement of the ADMGs in the appropriate control and test sites. Following this, gingival biopsies were procured at three different time intervals of 15, 45, and 90 days. There were significant differences in epithelial and Keratinized layer thickness among the three groups. This study concluded that there was no clear consensus on which graft material was superior but it gave an insight into the tissue response and wound healing process associated with the graft materials.

## 1. Introduction

The importance and role of the width of attached gingiva in periodontal health have been a constant source of debate and it remains insignificant in the presence of good oral hygiene [1], but a narrow band of attached gingiva can increase the risk of gingival inflammation and recession, along with impeding proper impression-making and the stability of orthodontic treatments [2,3,4]. However, it cannot be assumed that a thin band of keratinized gingiva is an indication for surgical intervention is warranted as there have been numerous clinical reports that state the absence of a keratinized zone of gingiva can correlate with the maintenance of periodontal health [5,6,7].

The necessity of an adequate band of keratinized gingiva around implants has not been well-reported, however, Han et al. [8] observed that augmentation procedures for the keratinized gingiva improved outcomes like effective plaque control, prevention of progression of gingival recession, and facilitate easier impression-making [9,10,11]. In orthodontic treatment, keratinized gingival augmentation may be warranted to prevent gingival recession that may occur with the movement of teeth. It can also be considered as a protective mechanism that can prevent marginal gingival inflammation in patients undergoing prosthodontic treatment [12,13,14]. Additionally, augmentation of keratinized gingiva may be indicated in cases of removable partial dentures and implants with high frenal attachment or reduced vestibular depth [15].

There are several techniques and graft materials that have been proposed for root coverage and augmentation of keratinized gingiva. These can be autografts like free gingival grafts (FGG), sub-epithelial connective tissue grafts (SCTG), or allografts like acellular dermal matrix graft (ADMG). In 1985, Langer and Langer proposed SCTG [16] and for decades it was touted as the ‘gold standard’ graft material because of its high success rate and predictability in augmenting the width of attached gingiva and root coverage [17]. The disadvantages of SCTG include a second surgical site for donor tissue, delayed healing and morbidity in the donor site, post-operative bleeding and discomfort for the patients. Even though the contention by Langer and Langer [16] was that the SCTG donor site would be closed when compared to FGG and thereby making it less uncomfortable, but it has been shown to increase the morbidity in the treated patients. The other limitations of SCTG include postoperative pain, haemorrhage, and necrosis [18]. Initially, allografts like preserved sclera, dura mater, and membranes (absorbable and non-absorbable) were used to replace autografts [19,20,21]. But more recently, it was reported that ADMG allograft could be used in mucogingival procedures to overcome the disadvantages of SCTG [22,23]. 

ADMGs were originally utilized for the treatment of full-thickness burn wounds in 1992, but they were subsequently introduced in periodontal surgery in the year 1994 as a substitute to FGG [24]. It can be obtained aseptically from the skin of the donor where the tissues undergo processing to remove the epidermal layer and the cellular components of the dermal layer i.e., the target of a graft rejection response. Also, the processing ensured the maintenance of the basement membrane and extracellular matrix. It has been utilized in various periodontal procedures like mucogingival surgery to augment the width of attached gingiva around natural teeth or implants, and gingival recession [25,26,27]. 

Human-derived ADMG (dADMG) was the first to be introduced and it has the longest referenced in the existing literature even though there have been other ADMGs in the market from allogenic and xenogenic sources [28,29,30]. Porcine-derived ADMG (mADMG), an acellular dermal collagen matrix where the porcine dermis undergoes processing for the removal of cells, non-collagenous proteins as well as immunogenic components [31]. Clinical reports with mADMG have shown that these grafts tend to improve the buccal soft tissue profile and the width of keratinized gingiva around implants [32,33]. An ideal ADMG should be recognized by the host, repopulated by the cells of the host, and promote re-vascularization when affixed with the host tissue. Thus, the processing steps in ADMG remain pivotal in achieving these desirable outcomes. The steps include separation of tissues through mechanical means, decellularization, dehydration, or lyophilization achieved through freeze-drying, and sterilization [30]. It should be duly noted that each of these steps can affect the architecture of the ADMG and ultimately, the host response to the graft material [34]. 

The framework of ADMG that is immunologically inert facilitates the migration of fibroblasts and revascularization when incorporated into the host tissues. A histological study on ADMG performed in a dog model observed that at 4 weeks, there was collagen formation with blood vessels penetrating the connective tissue and at 8 weeks, there was an increased penetration of blood vessels into the connective tissue and collagen bundles in various directions. There was an integration of the connective tissue and ADMG into a single vascularized structure, suggestive of complete incorporation of the graft at the 12-week mark [35]. To date, there are limited histological studies in the existing literature on allografts and this study was the first to compare two dAMGs graft materials. This animal model histomorphometric study aimed to compare these two grafts based on histological examination of gingival biopsies through parameters like, cellularity, angiogenesis, epithelial thickness, Keratinized layer thickness, matrix resorption, and inflammatory infiltrate with follow-up intervals at 15, 45, and 90 days.

## 2. Materials and Methods

The animal model study was carried out in female white mini pigs (*Susscrofa domestic*; n = 9) that was a cross of Landrace breed and Large white and they were approximately 3 months old weighing 22–25 kg and without periodontal disease. All protocols in this study were approved by the Ethics Committee and Animal Welfare of the University Hospital of Puerta de Hierro, Majadahonda, Madrid (n°: 017/2013) and the procedures regarding the handling and care of experimental animals complied with the European (Directive 2010.63.EU) and Spanish (RD 53/2013) regulations. A negative control group of gingival healing by secondary intention was utilized in this study.

### 2.1. Pre-Surgical Procedure

Prior to the surgical procedure, the mini pigs underwent overnight fasting and were anaesthetized using intramuscular injection of medetomidine (0.01 mg/kg, Medeson^®^, Uranus Vet, Barcelona, Spain), ketamine (5 mg/kg and 50 mg/mL, Ketalar^®^, Pfizer, New York, NY, USA), midazolam (0.2 mg/kg, Combino Pharm 10 mL, Barcelona, Spain), and atropine (0.02 mg/kg, Atropina B Braun^®^ 0.5 mL, B Braun Medical, Barcelona, Spain). Also, propofol (1 mg/kg, Propofol Fresenius^®^ 10 mg/mL, Fresenius Kabi Austria, Graz, Austria) was administered intravenously, which was followed by oxygen-isoflurane inhalation through endotracheal intubation. The monitoring of vital stats was performed by pulse oximetry and an electrocardiogram (ECG). Atipamezole (Antisedan^®^, Orion Corporation, Espoo, Finland) was used to reverse the effects of medetomidine at the end of the surgical procedure. 

### 2.2. Surgical Intervention

**Figure 1:**
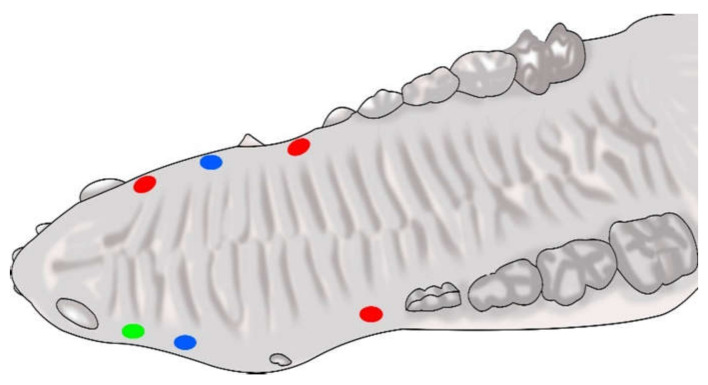
Schematic drawing of one of the pigs on the day of surgery showing: A green circle corresponding to a surgical site that will be left to gingival healing with secondary intention; two blue circles corresponding to surgical sites on which mADMG was subsequently placed; three red circles corresponding to surgical sites on which dADMG was subsequently placed.

**Figure 2:**
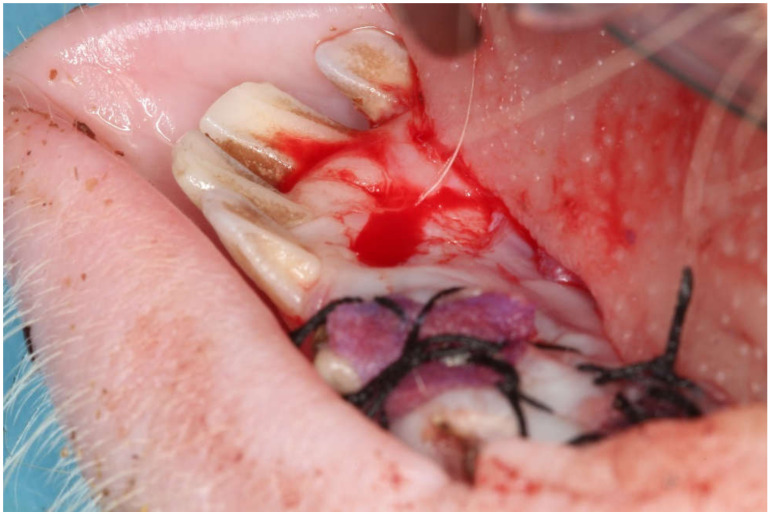
Post-surgical photograph showing: one surgical site to be allowed to heal by second intention; two surgical sites on which ADMGs have been placed randomly and sutured afterwards.

A single operator carried out the entire surgical protocol and local anaesthesia at the surgical site was achieved through injection of 4% articaine with 1:100,000 epinephrine (Ultracain^®^, Hoechst, Frankfurt, Germany). The surgical technique involved the creation of 5 mm diameter beds of keratinized tissue using a surgical punch on the edentulous areas resulting in the removal of epithelium and exposure of the underlying connective tissue. In each animal, the surgical sites were located in all four quadrants with 12 test areas for dADMG, eight areas for mADMG and two control areas. The test sites were grafted with dADMG (Osteobiol Derma^®^, Tecnoss^®^ Dental S.R.L., Giaveno, Italy), and mADMG (Mucoderm^®^, Botiss biomaterials GmbH, Zossen, Germany) whereas control sites were surgical beds that were left to heal by secondary intention (Figure 1). The placement of both the ADMGs took place without further cross-linking and it was fixed at the centre of the bed using a 2 mm micro-screw (Sweden & Martina^®^, Due Carrare, Italy). A crossed horizontal external suture was performed with a braided 5.0 single-thread silk suture and cutting needle. (Sweden & Martina^®^) held using a Castroviejo needle holder (Hu-Friedy^®^, Milano, Italy) and 41 Plain Adson Tissue Pliers (Hu-Friedy^®^, Milan, Italy). Figure 2. The samples were randomly procured from each animal at three different time intervals i.e., 15, 45, and 90 days for histological examination. 

### 2.3. Surgical Biopsy

The selection of the area for soft tissue biopsy followed a randomization process where cards that specify the different locations of the samples were divided into two envelopes and randomly allocated a scalpel blade (No 15c) mounted on a circular scalpel handle (Hu-Friedy^®^) and Adson forceps were used to procure the soft tissue biopsies. The soft tissue specimen would then be transferred into a canister specifically designed for this purpose and labelled with an identification code for each biopsy.

### 2.4. Euthanasia

Following the procurement of the gingival biopsies, controlled and regulated slaughter of the experimental animals was carried out by a veterinarian employing an overdose of intravenous sodium pentobarbital (Pfizer). The animals were previously sedated with 0.01 mg/kg medetomidine hydrochloride (Medeson^®^, Uranus Vet) and 5 mg/kg ketamine (Ketalar^®^, Pfizer).

### 2.5. Histological Preparation

All the study samples underwent resection and fixed using buffered 3.7% formalin solution for 48 h. The specimens underwent dehydration with increasing ethanol concentrations (70°, 90° & 100°), subsequently infiltrated, and embedded in paraffin. A cut and ground machine (Leica^®^, Wetzler, Germany) was used to prepare the specimens with an average section thickness of 5 µm and coated with silane, incubated at 56 °C for 24 h and then rehydrated with decreasing concentrations of ethanol. All the sections were stained using hematoxylin-eosin staining (Dako^®^, Santa Clara, CA, USA), mounted with DPX mountant (Vector Laboratories, Burlingame, CA, USA) and protected with a coverslip for evaluation under a light microscope (Olympus^®^ BX41, Barcelona, Spain) equipped with image analysis software (Image-Pro Plus^®^, Infaimon, Madrid, Spain). The assessed variables in this study include Keratinized layer thickness, epithelial thickness at the three-time intervals (15, 45, 90 days) followed by matrix resorption by fluorescence quenching at 90 days and acellular matrix resorption by quantifying areas at 90 days. 

### 2.6. Laboratory Process

A more detailed explanation regarding the laboratory steps in this histomorphometric study is given below.

#### 2.6.1. Matrix Staining

The ADMG matrices were immersed in 0.9% sodium chloride (NaCl) for 15 min under sterile conditions at room temperature. According to the modified protocol of Artzi et al. [36] the matrices were washed in a bicarbonate buffer (0.2 M, pH −8.3) for 5 min and incubated for 8 h at room temperature in Texas solution Red-X, succinimidyl ester 0.1 mg/mL (CF^TM^640R^®^, Sigma Aldrich, St. Louis, MO, USA). The matrices were previously solubilized in dimethylsulfoxide and immersed in conjugation buffer to allow for bonding at the covalent ends of the collagen chain or the esters of the pigment molecule. The unbound pigments were removed by washing with phosphate buffer for 3–5 min and the matrices were balanced at 0.9% NaCl before placement in the host tissues.

#### 2.6.2. Histomorphometric Analysis

**Figure 3:**
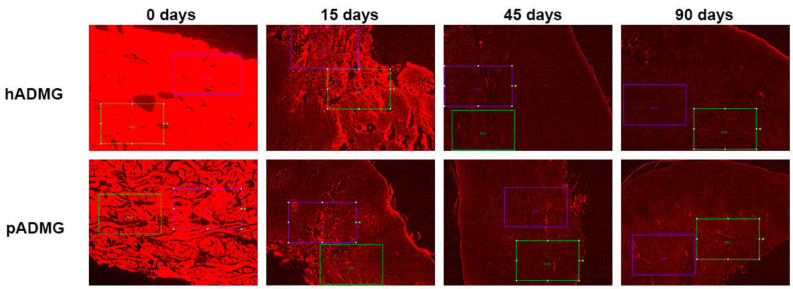
Fluorescence images at different times for the different ADMGs. Note the tracing of the analysis areas (100×).

The samples were observed using an optical light field microscope (BX41, Olympus, Barcelona, Spain) with an added image analysis software (Image-Pro Plus^®^, Infaimon, Madrid, Spain). Five different cuts of each sample were made and micrographs were taken on which the measurements for the Keratinized layer and depth of epithelial crests were made. On the same images, two areas were established on the connective tissue (35,000 µm^2^) for counting the number of cells (de novo cellularity) and areas of about 0.4 mm^2^ were used for counting the number of vessels along with their diameter. The numbers of inflammatory cells was estimated based on the resorption of implanted tissue that were observed in 0.5 mm^2^ areas to a relative percentage depending on whether the inflammatory acellular tissues were occupying those areas. The angiogenesis or the formation of new blood vessels in the graft was assessed by measuring the number of new blood vessels in the sample, the vessel size, followed by the minimum and maximum size of the vessel. The measurement of fluorescence was taken from the software for image analysis on undyed cuts of the samples (Figure 3).

### 2.7. Statistical Analysis

Statistical tests were performed using the SPSS Macros software (IBM Corp., New York, NY, USA). The values obtained were subjected to normality tests such as Kolmogrov–Smirnov, and Shapiro–Wilk’s tests, and the resultant data showed that it followed a parametric distribution. Further comparisons of the outcome parameters at three-time intervals (15, 45, 90 days) for each of the graft material was analysed using one-way ANOVA test. A *p* < 0.05 was considered as statistically significant and *p* < 0.001 was deemed as ‘highly significant’. 

## 3. Results

### 3.1. Epithelial Thickness

The mean epithelial thickness for dADMG was 281.49 ± 63.66 μm, whereas the mADMG showed a thickness of 279.56 ± 72.27 μm with the control group having a thickness of 236.70 ± 82.94 μm at the end of the 90-day follow-up (Table 1). A one-way ANOVA test within the three groups showed that there were statistically significant differences in epithelial thickness over the three different follow-up periods with a *p*-value of 0.001 (Table 2). Also, the differences in epithelial thickness were marked in each of the follow-up periods of 15 d, 45 d, and 90 d among the three study groups with a *p*-value of 0.001 (Table 3).

### 3.2. Angiogenesis

**Figure 4:**
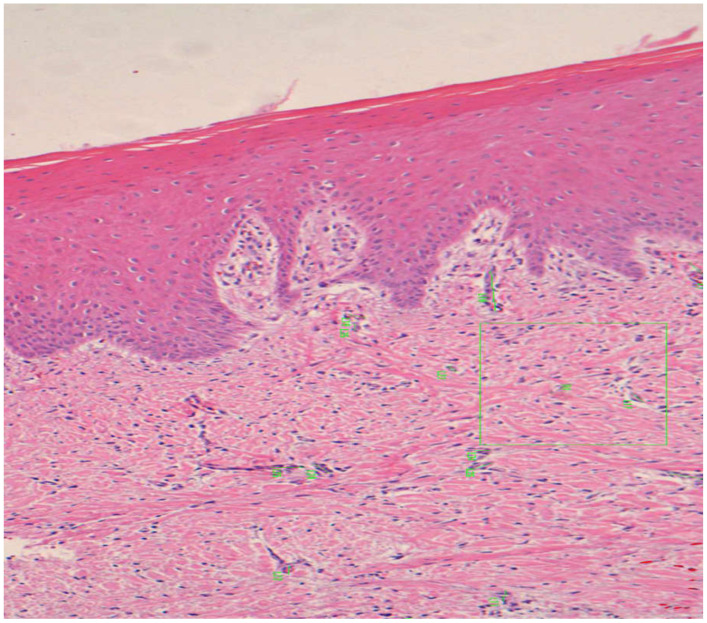
Detail of mADMG^®^ biopsy at 45 days showing the marking (green) of the vessels for measurement and counting (100×).

Figure 4 The average number of new vessels in dADMG graft increased from 16.43 to 24.16 over the follow-up period, however, there was a slight decrease in mADMG at 45 days, but it was the highest at the end of the follow-up period with a mean of 31.55 at 90 days. The mean vessel size was the highest in mADMG graft at 45 days with 40.41 ± 25.14, and the least vessel size was observed in dADMG graft at the same-follow up period with 27.37 ± 15.27 with the control group averages between 24.64 ± 10.24 and 38.34 ± 28.91 (Table 1). The comparison using one-way ANOVA within the three groups showed that there was a statistically significant difference in the vessel size over the follow-up period of 90 days (*p*-value −0.001) (Table 2). It was also observed that the differences were apparent in 15d (F-value −44.07), 45d (F-value −112.41), and 90d (F-value −16.33) among the three groups with *p*-value of 0.001 with high statistical significance (Table 3).

### 3.3. Keratinized Layer Thickness

There is an increase in mean keratinized layer thickness in mADMG from 18.01 ± 6.19 μm to 21.34 ± 7.95 μm over the follow-up period of 90 days, whereas there is a reduction in the dADMG graft over the follow-up period from 25.27 ± 11.04 μm to 19.46 ± 4.28 μm. The control group averaged between 19.57 ± 4.60 and 23.95 ± 6.64 over the follow-up period of 90 days (Table 1). One-way ANOVA test results show that there were significant differences in the Keratinized layer thickness within each of the study groups over the follow-up period of 15–90 days with *p*-values of 0.001; 0.005 (Table 2). Also, it was observed that the mean keratinized layer thickness showed highly statistically significant differences among the three study groups at 15d (F-value −21.65), 45d (F-value −9.82), and 90d (F-value −14.67) (Table 3).

### 3.4. Cellularity

**Figure 5:**
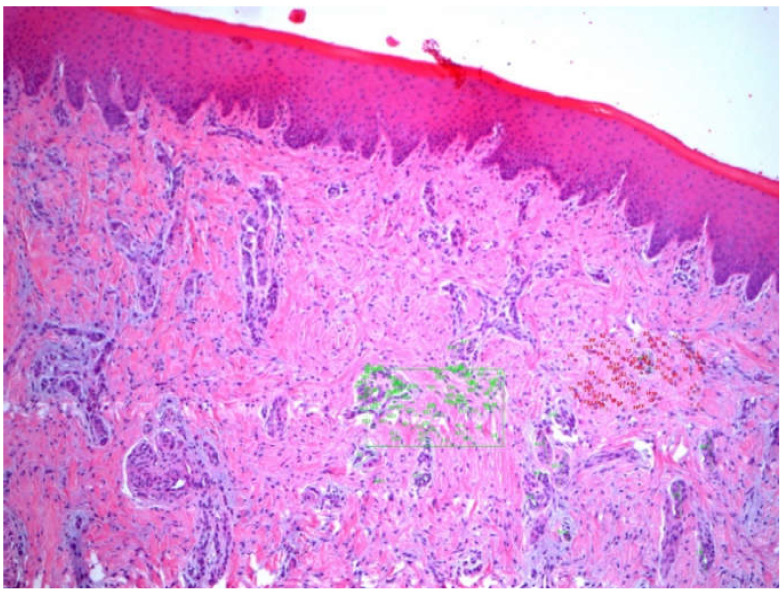
Two areas of 35,000 µm^2^ (green and red) on which the cells present were counted can be seen (100×).

The acellular matrix present in the graft was assessed as the cellular count was estimated and it was the highest in dADMG graft at 45 days (96.95 ± 36.33), whereas it was 80.52 ± 33.62 at the same time interval with mADMG and control group averaged 58.62 ± 15.04 in the same time period (Table 1) (Figure 5). A one-way ANOVA test showed that there were significant differences in cellularity in dADMG and the control groups over the follow-time period of 90 days (*p*-value 0.001), but mADMG did not show a significant difference over the same period, with a *p*-value of 0.23 (Table 2). The cellularity among the three groups was significantly different at the 15th day (*p*-value 0.02) and high significance at the 45th day (*p*-value 0.001) (Table 3). 

### 3.5. Inflammatory Infiltrate

**Figure 6:**
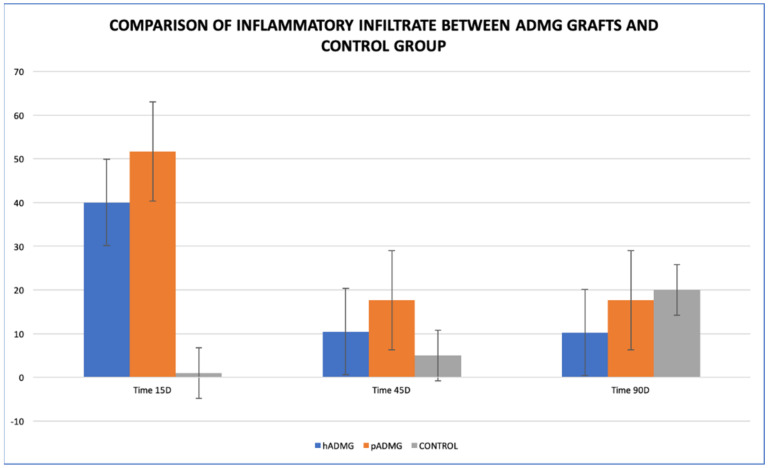
Comparison of inflammatory infiltrates between dADMG, mADMG grafts and Control Group.

**Figure 7:**
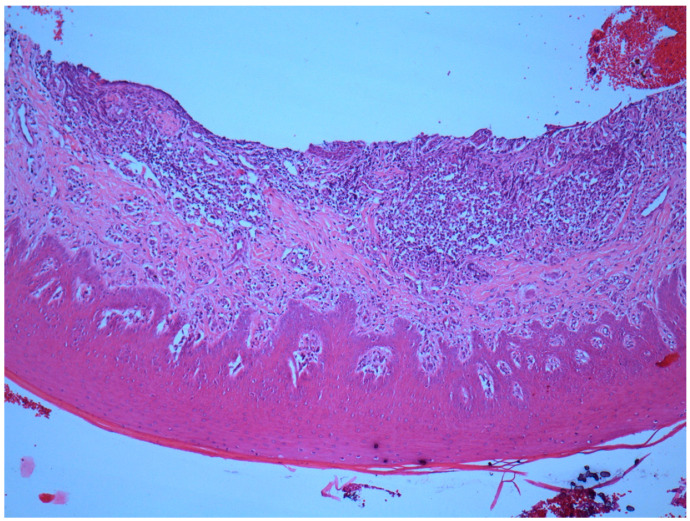
Control group (wound healing by second intention) biopsied after 90 days with very marked infiltration (10×).

There was increased inflammatory infiltrate in both the grafts at 15-days post-surgical intervention with mean values of 40 and 51.66, respectively, but the control group had a mean value of 1 during the same period. There was a decline in the inflammatory infiltrate over 45 and 90 days of follow-up in both the allografts, but it increased in the control group (Table 1) (Figure 6 and Figure 7). One-way ANOVA showed that only significant differences were present in dADMG over the follow-up period (*p*-value 0.015), whereas mADMG and control groups did not have significant differences in the inflammatory infiltrate over the follow-up period with *p*-values of 0.19 and 0.38 respectively (Table 2). Although there were differences between the inflammatory infiltrate among the three study groups, the differences were not statistically significant at 15 days, 45 days, and 90 days follow-up (Table 3).

### 3.6. Matrix Resorption

**Figure 8:**
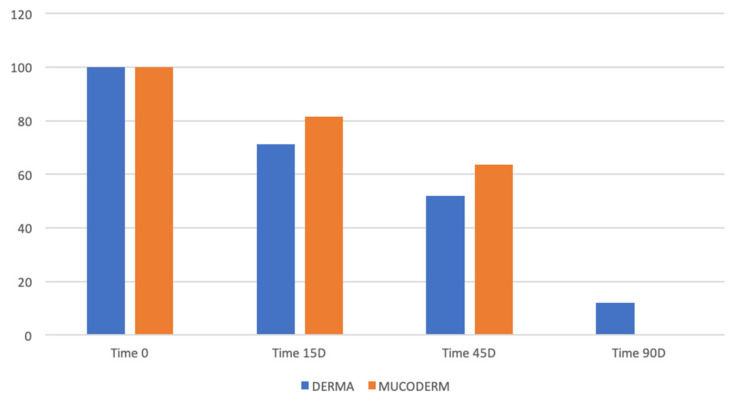
Comparison of matrix resorption between dADMG and mADMG grafts.

The matrix resorption was the highest in both the allografts at 15 days with mean values of 71.25 and 81.66 for dADMG and mADMG respectively. The average values reduced gradually over the follow-up period of 45 and 90 days (Table 1) (Figure 8).

## 4. Discussion

ADMG has been used as a substitute for soft tissue grafts in reconstructive and plastic surgeries. An ideal ADMG would be cost-effective, immunologically inert, and vascularize immediately and the latter characteristics are mutually exclusive as a coordinated immune response is essential for neovascularization [37]. In periodontal surgery, it has been considered as an alternative to autografts in mucogingival surgical procedures aimed for root coverage and increasing the width of the keratinized gingiva [38]. The mechanism of action of ADMG involves the provision of a natural scaffold made of collagen that will eventually be replaced by host collagen tissue. It forms a three-dimensional scaffold that allows for the selective repopulation of fibroblasts, ingrowth of new blood vessels, and epithelial proliferation from the surrounding tissues. A meta-analysis was conducted by Gapski et al. [23] in 2005 to assess ADMG-based mucogingival surgical procedures with other regularly utilized grafts such as FGG and CTG for root coverage and increase in the width of keratinized gingiva. It showed that there was increased keratinization with SCTG when compared ADMG, although not statistically significant. It was observed that the conclusions of the meta-analysis were tentative as there were limitations like weak study design and bias in reporting of trials [23]. 

There is limited data in the existing literature on the healing outcomes with ADMG although there are case reports where histological data has been provided [35,39]. In those reports, the ADMG was placed on a tooth with localized gingival recession and a hopeless prognosis and that may not be a perfect model to assess wound healing in these grafts [40]. Also, there have been studies where histological comparisons between ADMGs have been performed in plastic and reconstructive surgery, but there is no such data available for periodontal surgery [35]. A study conducted by Núñez et al. [39] showed that the mean gingival thickness with dADMG was around 1.46 ± 0.11 mm at the end of 3 months and similar results were obtained in a study by Sallum et al., [41] with a reported thickness of 1.63 ± 0.28 mm [39,41]. Histological analysis of connective tissue grafted with mADMG and SCTG in beagle dogs revealed that the thickness was 1.06 ± 0.27 mm and 1.32 ± 0.44 mm respectively [42]. In this study, it was observed that the epithelial thickness for dADMG was 281.49 ± 63.66 μm, 279.56 ± 72.27 μm for mADMG and 236.70 ± 82.94 μm for the control group at the end of 90 days’ follow-up. Despite the impossibility of direct comparison with other data yielded by the previously cited authors due to different experimental designs, from a histological point of view, there are significant differences between various biomaterials, however, the clinical implication of such variation in epithelial gain is more questionable. Based on this, and the difficulty of performing such studies in human models due to ethical issues, the comparison between these and other studies using various animal models should be performed in a complementary, rather than the comparative manner, to provide insights into the mechanisms of periodontal diseases and responses, as no animal model accurately represents tissue architecture and human healing processes [43].

The width of keratinized gingiva plays an important role in protection against various types of insults and it offers resistance to gingival inflammation and recession and hence mucogingival surgical techniques are dedicated to augmenting this zone around natural teeth or implants for that purpose. In a human study conducted by Wei et al., [44] the histological findings were compared between dADMG and FGG and it was observed that the degree of keratinization varied across the ADMG between sites as well as patients. It had features of ortho, para, and non-keratinized epithelium and also co-expression of para and non-keratinized epithelium rather than homogenous orthokeratinization [44]. In this study, there was a gradual reduction in the keratinized layer thickness with dADMG over the follow-up period, whereas mADMG showed a gradual increase that was statistically significant. There were significant differences in the keratinized layer thickness among the three study groups at each time interval of follow-up and it was highly significant. Wei’s findings on connective tissue demonstrated that it was devoid of cells and had had a higher percentage of collagen and elastin fibres. In this study, similar findings were observed where there was a decreasing trend in the cellularity over the period of 90 days, suggestive of increased extracellular matrix formation [44]. 

A study conducted by Suárez-López et al. [45] compared the histological outcomes between porcine-derived ADMG and collagen-based matrix (CBM) for the treatment of gingival recession in beagle dogs and the follow-up period included 2, 6, and 10 weeks’ post-surgical intervention. The degree of vascularization was calculated from the percentage of blood vessels in the region of interest (ROI) and there was reduced vascularization in CBM when compared to ADMG at all time intervals and the changes were statistically significant [44,45]. In this study, there was a consistent increase in the number of blood vessels per sample in both the allografts with the highest average in mADMG (collagen-based matrix) and in concordance with the observations from the abovementioned study. A study has been performed with biofunctionalization of mADMG along with autologous platelet concentrates like platelet-rich fibrin (PRF) and it was observed that there was an increased growth factor (vascular endothelial growth factor or VEGF), transforming growth factor-β [(TGF-β)] release and improved angiogenic potential after 7 days when compared to only PRF [46]. A similar functionalization pilot study on ADMGs with growth factors [fibroblast growth factor or FGF-2), TGF-β1, platelet-derived growth factor (PDGF-BB), and bone morphogenetic protein (BMP-2)] showed significant promise with improved adsorption and release of the growth factors [47]. Future clinical trials on the application of ADMGs along with platelet concentrates or growth factors in periodontal plastic surgical procedures are warranted.

It is vital to determine the immuno-inflammatory reaction of an allogenic material with host tissues. According to Núñez et al., there was no sign of chronic inflammatory reaction suggestive of an absence of foreign body reaction in ADMG group [39]. Wei et al. suggested that it is not uncommon to find inflammatory infiltrate in clinically healthy gingiva but there was increased inflammatory infiltrate in the ADMG group that could imply either a foreign body reaction or extended reaction to the process of wound healing [43,44]. These findings were contradictory to the observations made by Suárez-López et al. [44,45], where there was neither chronic inflammatory infiltrate nor necrotic tissue suggestive of favorable tissue reaction. In this study, there were no significant differences in the inflammatory infiltrate when compared among the three groups over each of the time intervals of follow-up. Interestingly, a progressive increase of inflammatory infiltration was observed over time, being more prominent at 90 days. These results are contradictory with the ones presented by Mak et al. [48], where when analyzing wounds healed by second intention in oral mucosa of Duroc pig models, inflammatory response increased during the first 14 days, to start a progressive decrease afterwards. To our knowledge, no study using the same animal model has evaluated this variable over a period of time of 90 days, in this sense, more studies are necessary to realize a proper comparison of this outcome.

This histomorphometric study was conducted on a porcine animal model employing porcine-derived ADMGs for the augmentation of the width of keratinized gingiva and comparisons were established with a negative control group that utilized gingival healing with secondary intention over a follow-up period of 90 days. A conclusion of better graft material could not be drawn from this study as both the ADMGs successfully integrated with the host tissue and there were improved outcomes with no adverse reactions apart from inflammatory changes. The limitation of this animal model study includes a short follow-up period of 90 days and a longer follow-up may have provided more insights into the clinical results. However, this study aimed to assess the tissue response and healing process in these ADMGs rather than to assess the ideal graft material for augmentation of keratinized gingiva. There are numerous clinical scenarios for the application of these graft materials and future research must explore the combination of surgical technique and ideal graft material to be used.

## 5. Conclusions

This study concluded that there were significant differences between the three study groups in terms of epithelial and keratinized layer thickness, and angiogenesis. Both the allografts did not present with significant inflammatory infiltrate and did not show any graft rejection. Further studies are warranted to explore the ideal graft material and to determine the appropriate clinical technique to be used in conjunction with the graft material. 

## Figures and Tables

**Table 1:** Descriptive statistics of the outcome parameters between admg grafts and control groups during follow-up intervals.

Variables	dADMG			mADMG			Control		
	15d (n = 39)	45d (n = 20)	90d (n = 25)	15d (n = 29	45d (n = 18)	90d (n = 20)	15d (n = 5)	45d (n = 8)	90d (n = 10)
Epithelial Thickness (μm)	438.28 ± 159.41	270.75 ± 75.19	281.49 ± 63.66	330.10 ± 139.71	347.15 ± 65.69	279.56 ± 72.27	267.74 ± 63.26	489.10 ± 148.89	236.70 ± 82.94
Angiogenesis (Vessel Size)	34.15 ± 18.76	27.37 ± 15.27	33.45 ± 21.72	30.15 ± 17.35	40.41 ± 25.14	31.33 ± 22.05	24.64 ± 10.24	39.76 ± 24.72	38.34 ± 28.91
Keratinized layer thickness (μm)	25.27 ± 11.04	23.60 ± 6.13	19.46 ± 4.28	18.01 ± 6.19	20.07 ± 6.58	21.34 ± 7.95	19.57 ± 4.60	23.95 ± 6.64	17.25 ± 8.71
Cellularity	76.85 ± 20.74	96.95 ± 36.33	58.42 ± 35.77	73.77 ± 22.88	80.52 ± 33.62	69.65 ± 28.95	94.40 ± 12.99	58.62 ± 15.04	73.20 ± 27.86
Inflammatory Infiltrate	40.00 ± 14.14	10.50 ± 6.13	10.25 ± 16.50	51.66 ± 31.75	17.66 ± 14.64	17.66 ± 19.85	1.00 ± 0.01	5.00 ± 0.001	20.00 ± 21.21
Matrix resorption	71.25	52	12	81.66	63.75	0	-	-	-

dADMG: human derived acellular dermal matrix graft; mADMG: porcine derived acellular dermal matrix graft.

**Table 2:** one-way ANOVA for the comparison of changes in outcome parameters during the follow-up time intervals between ADMG grafts and control groups.

Outcome Variables	Groups	ANOVA F Value			*p*-Value
		15d	45d	90d	
Epithelial Thickness	dADMG	147.149			0.001 **
	mADMG	23.193			0.001 **
	Control	139.392			0.001 **
Angiogenesis	dADMG	19.66			0.001 **
	mADMG	32.77			0.001 **
	Control	12.89			0.001 **
Keratinized layer Thickness	dADMG	37.42			0.001 **
	mADMG	5.43			0.005 *
	Control	24.81			0.001 **
Cellularity	dADMG	18.51			0.001 **
	mADMG	1.46			0.23
	Control	8.61			0.001 **
Inflammatory infiltrate	dADMG	6.88			0.015 *
	mADMG	2.14			0.19
	Control	1.33			0.38

dADMG: human derived acellular dermal matrix graft; mADMG: porcine derived acellular dermal matrix graft. * *p*-value is <0.05; ** *p*-value is <0.001.

**Table 3:** one-way ANOVA for the comparison of changes in outcome parameters between ADMG grafts and control groups at various periods.

Outcome Variables	Time Period	ANOVA F Value			*p*-Value
		dADMG	mADMG	Control	
Epithelial Thickness	15 days	44.07			0.001 **
	45 days	112.41			0.001 **
	90 days	16.33			0.001 **
Angiogenesis	15 days	16.31			0.001 **
	45 days	47.34			0.001 **
	90 days	7.30			0.001 **
Keratinized layer Thickness	15 days	21.65			0.001 **
	45 days	9.82			0.001 **
	90 days	14.67			0.001 **
Cellularity	15 days	4.02			0.02 *
	45 days	8.21			0.001 **
	90 days	2.12			0.12
Inflammatory infiltrate	15 days	3.72			0.08
	45 days	1.12			0.38
	90 days	0.23			0.79

dADMG: human derived acellular dermal matrix graft; mADMG: porcine derived acellular dermal matrix graft. * *p*-value < 0.05; ** *p*-value < 0.001.
